# Oat species and interspecific amphiploids show predominance of diploid nuclei in the syncytial endosperm

**DOI:** 10.1007/s13353-023-00798-0

**Published:** 2023-11-07

**Authors:** Paulina Tomaszewska, Romuald Kosina

**Affiliations:** 1 Department of Genetics and Cell Physiology, Faculty of Biological Sciences, University of Wrocław, Kanonia 6/8, 50-328 Wrocław, Poland; 2 Department of Genetics and Genome Biology, University of Leicester, 1LE 7RH, Leicester, UK; 3 Faculty of Biological Sciences, University of Wrocław 51-148, Przybyszewskiego, 63 Wrocław, Poland

**Keywords:** *Avena*, Endosperm, Diploid syncytium, Ploidy levels vs anomalies, Correlation of traits, Ordination analysis

## Abstract

Apart from apomictic types, the *Polygonum-*type eight-nuclear embryo sac is considered to be dominant in grasses. A triploid endosperm is formed as a result of double fertilisation. This study showed, for the first time, the dominance of diploid nuclei in the syncytial stage of the central cell of embryo sac in oat species and amphiploids. The dominance of diploid nuclei, which were the basis for the formation of polyploid nuclei, was weaker in amphiploids due to aneuploid events. The genomic in situ hybridisation method applied in the study did not distinguish the maternal and paternal haploid nuclei of embryo sac. However, this method demonstrated the lack of a set of genomes of one haploid nucleus. Embryological analyses of the initial stages of oat endosperm development revealed a fertilised egg cell, and two polar nuclei differing in size. It can be assumed that the formation of diploid oat endosperm occurred after the fusion of one polar nucleus and the nucleus of a male gamete, while the second polar nucleus gave rise to 1*n* nuclei. The levels of ploidy of syncytial nuclei were not influenced by both aneuploid events and correlated with pollen developmental anomalies. The differences in the analysed cytogenetic events distinguished amphiploids and their parental species in the ordination space.

## Introduction

Embryo sacs of Angiospermae constitute a broad spectrum of structural types. The *Polygonum*-type embryo sac occurs in the majority of flowering plants (Willemse and Went 1984) and is treated almost as a paradigm in the field of botany. It is known that grasses, including cereals, develop this type of embryo sac. In their study, Friedman and Ryerson (2009) showed that a four-nucleate module can be distinguished in the development of the embryo sac, both in basal angiosperms, such as *Amborella*, and in monocots with the *Polygonum*-type. Finally, the fusion of the polar nuclei from two modules results in the formation of a triploid endosperm. In cereals, a dominant 3C-level polyploidy in a central cell of embryo sac is maintained throughout the development, up to the ripe endosperm tissue, for example in *Zea* or *Hordeum vulgare*. Fluctuations can be seen only between this level and its multiplications 6C, 12C, and 24C (Nguyen et al. 2007; Nowicka et al. 2021). However, the pattern of DNA multiplication was found to remain stable in *Zea mays* hybrids, as it was altered indicating the maternal quantitative effect (Kowles et al. 1997).

In the *Avena* species and amphiploids, Tomaszewska (2017) found that the endosperm in the syncytial stage varied from that observed in the typical *Polygonum*-type embryo sac. Additionally, some deviation from the *Polygonum* development was noted in *Avena fatua*. In addition to the initial 3C and 6C levels, the 2C level appeared in the mature aleurone layer, which is the youngest tissue of the endosperm (Maherchandani and Naylor 1971). This gives rise to the speculation whether the ‘*Polygonum* thinking’ is applicable to cereals, especially the genus *Avena*.

Several detailed studies conducted on *Arabidopsis* have proven that many genes likely have an impact on the development of embryo sac and specification of central cell fate. Certain genes, such as *CKI1* (histidine kinase), can be expressed during female gametophyte development, restricting the fusion of the polar nuclei and interacting with the antipodals activity (Li and Yang 2020). The occurrence of the *Polygonum-*type embryo sac was unstable in the sexually reproducing *Maxillaria* orchid, where micropylar and chalazal nuclei remain unfused (Kolomeitseva et al. 2022). However, studies show that in *Tulipa* sp. with the tetrasporic *Fritillaria*-type embryo sac and a pentaploid endosperm, the fusion of micropylar (one maternal) and sperm (one paternal) nuclei results in the formation of a diploid endosperm (Mizuochi et al. 2009). These indicate that there are several variants of endosperm development within the same type of embryo sac.

Apomictic and autonomous development of embryo sac and endosperm, independent of fertilisation, can be observed in some plants, including grasses (Nogler 1984; Vinkenoog and Scott 2001). Apomixis, as a complex developmental process, results in the formation of an endosperm with an altered level of ploidy. A study showed the formation of multiporate pollen grains in apomictic plants (Ma et al. 2009). Such types of pollen grains have been found in the genus *Avena* in artificial amphiploids (Tomaszewska and Kosina 2022), which could suggest somehow the apomictic development of their embryo sacs.

Natural hybrids and increased ploidy levels are common in plants (Grant 1981). This phenomenon was also applied to obtain new types of crops, such as Triticale. In both somatic tissues and storage endosperm of plants, it is accompanied by cytogenetic disturbances, which manifest either as amplification or loss of DNA, or even as the elimination of whole genomes, especially in hybrids (Gvaladze et al. 2002; Tomaszewska and Kosina 2018). In *Avena*, such changes, in particular the high level of translocations with an asymmetric frequency between genomes, results in the differentiation of genomes (Tomaszewska et al. 2022), which highlights the unique cytogenetic status of oats among cereals.

Such specificity of the genus *Avena* became the basis for research on the level of endosperm ploidy in various species and artificial amphiploids as advanced breeding forms. The undertaken analyses concern storage tissue, which is important in human nutrition and whose development is conditioned by cytogenetic events (Kosina 2016; Tomaszewska and Kosina 2018). Therefore, the recognition of tissue variability at the cytogenetic level provides important information in plant breeding focused on its quality. The main objectives of this study were therefore to investigate (1) the ploidy level of syncytial endosperm in oat species and artificial amphiploids, (2) the initial stage of oat endosperm development, and (3) the correlation between the endosperm ploidy and aneuploidy and the anomalously developed pollens.

## Material and methods

### Plant materials

Species and amphiploids of oats used in the study are listed in Table 1. The investigated *Avena* species were classified based on the nomenclature adopted from https://npgsweb.ars-grin.gov/gringlobal/taxon/taxonomysearch.aspx (accessed on 2 December 2022) and http://www.theplantlist.org/ (accessed on 2 December 2022).

**Table 1:** Accessions of oat amphiploids and parental species used in the study

Amphiploids/parental species	Symbols used in figures	Accesion number	Donor	Origin	Determined ploidy level in roots	Expected chromosome number in 3*n* endosperm
*A. barbata* × *A. sativa* ssp. *nuda*	b/sn	CIav7903	NSGC	-	Octoploid	84 (12*x*)
*A. eriantha* × *A. sativa*	e/sa	PI458781	NSGC	-	Octoploid	84 (12*x*)
*A. barbata* × A. sativa*	b/sa	CIav7901	NSGC	-	Hexaploid	63 (9*x*)
*A. fatua* × A. sterilis*	f/ste	CIav9367	NSGC	-	Hexaploid	63 (9*x*)
*A. magna* × A. longiglumis*	m/l	CIav9364	NSGC	-	Hexaploid	63 (9*x*)
*A. abyssinica* × A. strigosa*	a/str	CIav7423	NSGC	-	Tetraploid	42 (6*x*)
*A. fatua* L.*	Af	-	R. Kosina	Poland	Hexaploid	63 (9*x*)
*A. sterilis* L.	Aste	PI311689	NSGC	Israel	Hexaploid	63 (9*x*)
*A. sativa* L.*	Asa	-	R. Kosina	Poland	Hexaploid	63 (9*x*)
*A. barbata* Pott ex Link	Ab	AVE1938	BAZ	Spain	Tetraploid	42 (6*x*)
*A. abyssinica* Hochst.	Aa	PI331373	NSGC	Ethiopia	Tetraploid	42 (6*x*)
*A. abyssinica* Hochst.*	-	14671	VIR	?	Tetraploid	42 (6*x*)
*A. magna* Murphy et Terrell*	Am	1786	VIR	Morocco	Tetraploid	42 (6*x*)
*A. magna* Murphy et Terrell*	-	CIav8331	NSGC	Morocco	Tetraploid	42 (6*x*)
*A. strigosa* Schreb.	Astr	51624	BAZ	Belgium	Diploid	21 (3*x*)
*A. longiglumis* Dur.	Al	PI367389	NSGC	Portugal	Diploid	21 (3*x*)
*A. eriantha* Dur.	Ae	CIav9051	NSGC	England	Diploid	21 (3*x*)

*Accessions of oats used for embryological analysis

*BAZ* Bundesanstalt für Züchtungsforschung an Kulturpflanzen, Braunschweig, Germany; *NSGC* National Small Grains Collection, Aberdeen, ID, USA; *VIR* Vavilov Institute of Plant Industry, St. Petersburg, Russia

The seeds of oats were germinated for 3 days on a moisturised filter paper at 25°C in the dark. Then, the seedlings were planted into pots filled containing a mixture of soil and sand in a ratio of 3:1. All the plants were grown under the same climatic conditions. Next, young kernels at the syncytial stage of endosperm development were harvested. Based on the morphology of the pistil and the degree of dryness of the stigma, the stage of endosperm development was defined. During five consecutive growing seasons, approximately a total of 200 developing seeds from each accession were collected from the spikelets at 2–4 days after pollination (DAP).

### Isolation and fixation of endosperm

Using a binocular microscope, embryo sacs were manually dissected from developing seeds. The peeled embryo sacs were then fixed in 96% ethanol–glacial acetic acid (3:1) fixative for 48 h at room temperature. This was followed by the fixation of embryo sacs in a freshly prepared fixative solution at 4°C.

### Preparation of endosperm chromosomes and genomic in situ hybridisation

Most cytogenetic endosperm analyses have thus far been performed on paraffin sections. However, in this study, the enzymatic maceration method was used to obtain better chromosome spreads. This technique involves squashing chromosomes between a slide and a coverslip by applying thumb pressure. The modified method of Schwarzacher and Heslop-Harrison (2000) was used for this purpose, which was originally developed for chromosome preparation from root tips. The fixed embryo sacs were rinsed in distilled water for 15 min, and then washed in 0.01 M citrate buffer three times for 5 min each. Subsequently, the embryo sacs were transferred to a maceration enzyme solution (0.3% cellulase, 0.3% pectolyase, 0.3% cytohelicase in 0.01M citrate buffer) and digested in a hybridisation oven (Biometra OV3) at 37°C for 45 min. They were then washed in 0.01 M citrate buffer three times for 5 min each. In the next step, the digested embryo sac was placed on a clean slide in a drop of 45% acetic acid for 1 min, and then cut open with a scalpel. Only the liquid content of the embryo sac was left on the slide, and the other tissues were discarded. The endosperm tissue was then squashed in 45% acetic acid under a coverslip by applying light thumb pressure. Finally, the slides were frozen in liquid nitrogen and air-dried. Genomic in situ hybridisation (GISH) was performed on endosperm as previously described by Tomaszewska and Kosina (2021), using genomic DNAs extracted from fresh leaves of *Avena eriantha* (C genome) and *Avena nuda* (A genome), labelled with tetramethylrhodamine-5-dUTP (red fluorescence) and digoxigenin-11-dUTP, respectively, as probes. The probe labelled with digoxigenin was detected with fluorescein isothiocyanate-conjugated sheep anti-digoxigenin antibody (green fluorescence). The slides were then counterstained with DAPI and analysed under Olympus BX-50 and BX-60 fluorescence microscopes (Hamburg, Germany). Images were taken with an Olympus E-520 camera (Olympus Imaging Europa GMBH, Hamburg, Germany).

### Embryological analysis

In order to analyse the initial stages of endosperm development, we used the 2,2′-thiodiethanol (TDE)-based tissue clearing protocol (Musielak et al. 2016). Fixed embryo sacs of various sizes were rinsed in 2X PBS (phosphate buffered saline, pH 7.4) three times for 5 min each. Subsequently, the embryo sacs were incubated in 20% and 40% glycerol in 50mM Tris pH 9.5 for 1h each at 4°C, followed by incubation in 50% TDE in 50mM Tris pH 9.5 supplemented with 50 μg mL^−1^ propidium iodide (PI) at 4°C for 12h. The embryo sacs were placed on a clean slide and mounted in 50% TDE in 50mM Tris pH 9.5. An Olympus FluoView FV1000 confocal laser scanning microscope was used to image embryo sacs. The images were recorded by employing the PlanApochromat ×10, ×20, and ×40 water immersion objectives. A total of 126 embryo sacs from 9 oat accessions (see Table 1) were analysed.

### Isolation of pollen grains

Pollen grains were collected during three consecutive vegetation seasons. The frequency of formation of micropollens and multiporate pollens was determined in oat species and amphiploids. Detailed studies on morphotypes and viability of pollen grains in oats have already been published (Tomaszewska and Kosina 2022).

### Correlation and numerical taxonomy analyses

A matrix of correlation coefficients and data for nonmetric multidimensional scaling (nmMDS) were obtained using Rohlf’s approach (Rohlf 1994).

## Results and discussion

### Variation of chromosome number in oat syncytial endosperm

The chromosome number variation in the cereal endosperm was larger than that observed in other vegetative tissues, which can be due to significant DNA amplification and aneuploidy events (Kaltsikes and Roupakias 1975; Nowicka et al. 2021). Our studies carried out on oat endosperm showed that amphiploids derived from hexaploid parents are characterised by greater variation in the number of chromosomes in this tissue compared to amphiploids derived from 2*x* and 4*x* parents. Among the oat species, *A.magna* (4*x*) and *A*. *eriantha* (2*x*) showed extreme variation in chromosome numbers. It was observed that amphiploids had a twofold larger range of chromosome numbers than parental species. The position of modal values for the 2*n* chromosome numbers indicates the left-hand skewness of distributions in most taxa. The only exceptions were *A*. *magna* (reverse-J shaped distribution) and *A*. *longiglumis* and *A*. *eriantha* (right-skewed distribution), while some showed nearly symmetric distributions (Table 2).

**Table 2:** Number of chromosomes observed in the syncytial endosperm of oat species and amphiploids

Amphiploids/parental species	Number of analysed metaphases	Number of chromosomes	rcn
*A. barbata* × *A. sativa* ssp. *nuda*	179	35, 36, 41, 42, 47, 51, 52, 54, 55, **56**, 57, 61, 104, hp	69
*A. eriantha* × *A. sativa*	201	28, 40, 42, 48, 49, 50, 52, 53, 54, 55, **56**, 104	76
*A. barbata* × *A. sativa*	85	21, 32, 36, 37, 38, **42**, 44, 48, 56, 63, hp	42
*A. fatua* × *A. sterilis*	203	25, 28, 30, 32, 35, 37, 38, 39, 40, 41, **42**, 61, 63, 84, hp	59
*A. magna* × *A. longiglumis*	116	39, 40, 41, **42**, 52, 63, hp	24
*A. abyssinica* × *A. strigosa*	164	14, 19, 21, 26, 27, **28**, 42, hp	28
Σ/*n* (for amphiploids)			49.7
*A. fatua*	174	21, 26, 28, 37, 40, 41+1r, **42**, 56	39
*A. sterilis*	141	41, **42**, 78, 84	43
*A. sativa*	91	36, 38, 40, **42**	6
*A. barbata*	91	26, **28**, 42	16
*A. abyssinica*	68	13, 14, 26, 27, **28**	15
*A. magna*	111	**28**, 42	14
*A. strigosa*	194	10, 11, 12, 13, **14**, 17, 19, 21, 23, 28, hp	18
*A. longiglumis*	49	12, **14**, 21, 28, 36, 38, 42, hp	30
*A. eriantha*	203	7, 12, 13, **14**, 14+1r, 17+1r, 18+1r, 20+2r, 21, 22, 28, 42, hp	35
Σ/*n* (for species)			24.0

2*n* numbers, based on the determined ploidy level in roots (see Table 1), are given in bold

*r* ring chromosome; *hp* hiperploidy; *rcn* range of chromosome number

The development of a *Polygonum-*type embryo sac with a triploid endosperm is common in Angiospermae (Willemse and Went 1984). At a later stage of endosperm development, DNA amplification runs from 3C to 6C and 12C up to 24C in grasses with their cultivated taxa, such as wheat or barley (Chojecki et al. 1986; Nowicka et al. 2021). There were much higher levels of ploidy in rice and sorghum, rising to over 96C in maize (Nguyen et al. 2007). In the oat syncytial endosperm, we observed nuclei with a high level of ploidy (indicated by the symbol ‘hp’ in Table 2), and the detailed values hidden therein would undoubtedly widen the range of chromosome numbers. We suppose that the fusion of two smaller nuclei, among others, can result in the formation of hp-type nuclei with a high and variable number of chromosomes. This is evidenced by the formation of giant nuclei in *Arabidopsis* (Baroux et al. 2004), and dumbbell nuclei in Triticale (Kaltsikes and Roupakias 1975), as well as in *Avena* species and amphiploids (Tomaszewska 2017). Our studies showed that, unlike wheat or barley (Chojecki et al. 1986; Nowicka et al. 2021), cytogenetic variability of endosperm is greater in the genus *Avena*, both in hybrids and in species. This may be due to the increase in cytogenetic variability by aneuploidy. In Table 3, the series of chromosome numbers refer to aneuploid states, which were more numerous in amphiploids than in species.

**Table 3:** Percentage of metaphases in the syncytial endosperm of different ploidy level

Amphiploid/parental species	1*n*	2*n*	3*n*	4*n*	6*n*	ap	hp
*A. barbata* × *A. sativa* ssp. *nuda*	0	57.14	0	0	0	40.82	2.04
*A. eriantha* × *A. sativa*	1.96	54.90	0	0	0	43.14	0
*A. barbata* × *A. sativa*	2.17	69.57	2.17	0	0	23.92	2.17
*A. fatua* × *A. sterilis*	1.04	64.58	2.08	1.04	0	30.22	1.04
*A. magna* × *A. longiglumis*	0	88.52	1.64	0	0	8.20	1.64
*A. abyssinica* × *A. strigosa*	1.64	59.02	14.75	0	0	22.95	1.64
Σ/*n* (for amphiploids)	1.14	65.62	3.44	0.17	0	28.21	1.42
*A. fatua*	6.52	80.43	0	0	0	13.05	0
*A. sterilis*	0	87.18	0	2.56	0	10.26	0
*A. sativa*	0	90.00	0	0	0	10.00	0
*A. barbata*	0	70.83	20.83	0	0	8.34	0
*A. abyssinica*	14.29	71.43	0	0	0	14.28	0
*A. magna*	0	95.00	5.00	0	0	0	0
*A. strigosa*	0	66.06	24.24	1.21	0	7.88	0.61
*A. longiglumis*	0	29.03	25.81	9.68	22.58	9.67	3.23
*A. eriantha*	0.68	71.92	17.12	0.68	0.68	8.24	0.68
Σ/*n* (for species)	2.39	73.54	10.33	1.57	2.58	9.08	0.50

1*n*…6*n* levels of ploidy, *ap* aneuploidy, *hp* hyperploidy

### Ploidy levels of oat syncytium

**Fig. 1:**
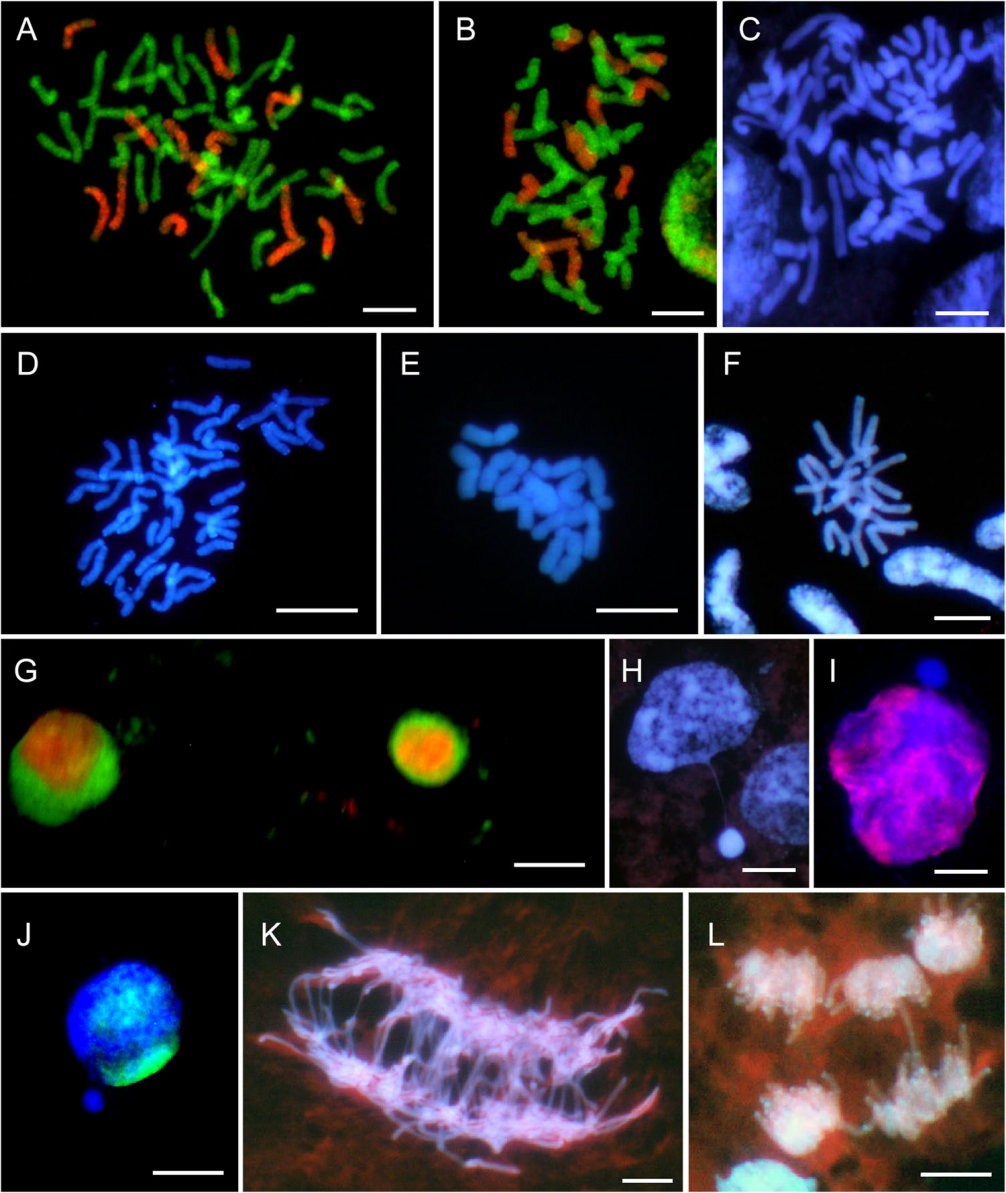
Data on 2*n* chromosome numbers and some cytogenetic disorders in the oat syncytial endosperm. **a** GISH metaphase in *A. barbata* × *A. sativa* ssp. nuda with 2*n* = 56; 14 red chromosomes of genome C, 42 green chromosomes of genomes A and D; there is a lack of genome B and 28 chromosomes of A/C/D genomes. **b** GISH metaphase in *A. barbata* × *A. sativa* with 2*n* = 42; 14 red chromosomes of genome C, 28 green chromosomes of genomes A and D; there is a lack of 21 chromosomes of A/C/D genomes. **c** DAPI metaphase in *A. eriantha* × *A. sativa* with 2*n* = 56. **d** DAPI metaphase in *A. fatua* × *A. sterilis* with 2*n* = 42. **e** DAPI metaphase in *A. strigosa* with 2*n* = 14. **f** DAPI metaphase in *A. eriantha* with 2*n* = 14. **g** GISH interphase nuclei in *A. sterilis* with green external A/D genomes and internal red C genome. **h** DAPI prophase nuclei with condensed micronucleus connected by a thread of chromatin with upper left nucleus in *A. eriantha* × *A. sativa*. **i** An early GISH prophase nucleus with an extruded micronucleus composed of A/D genomes in blue (genomes C, red/violet) in *A. barbata* × *A. sativa* ssp. nuda. **j** A GISH interphase-prophase nucleus in *A. abyssinica* with an extruded micronucleus composed of B genome (A genome in green). **k** DAPI anaphase with multiple bridges in *A. barbata* × *A. sativa*. **l** DAPI multipolar anaphases in *A. eriantha*. Scale bars = 10 μm

All the studied taxa showed a dominance of diploid nuclei (2*n*) in the syncytial endosperm (Table 3; Fig. 1a–f). Therefore, the phenomenon of diploid syncytium can be assumed for the whole *Avena* genus. Two species, *A. magna* and *A*. *longiglumis*, showed the greatest difference of dominance, with 95% of diploid nuclei and only 29.03%, respectively. Triploid nuclei were not numerous in amphiploids but were more frequent in tetraploid (*A*. *barbata*) and diploids (*A*. *strigosa*, *A*. *longiglumis*, and *A*. *eriantha*). Some uniformity of nuclei frequency was observed at higher levels of ploidy, as in the case of *A*. *longiglumis*, which were formed at the cost of the 2*n* nuclei. Hyperploidy and aneuploidy were threefold more frequent in amphiploids than in parental species. The highly polyploid nuclei were absent in 4*n* and 6*n* species. It was noted that *A*. *abyssinica* and *A*. *fatua* had a high percentage of 1*n* nuclei. No 3*n* nuclei have been recorded in these species, because they would just be formed from the union of 1*n* and 2*n*. The differences between taxa in the absence or presence of 4*n* or 6*n* nuclei indicate the intertaxa variation of the polyploidisation rate.

The GISH method enabled the identification of A/D and C genomes in the nuclei of the syncytial endosperm and showed a lack of one haploid set of genomes (Fig. 1a, b). Such analysis, however, did not allow to assess whether the nuclei of the syncytial endosperm lack chromosomes from the female polar nuclei or male sperm nuclei as they are genomically identical. Lange (1971) and Bennett et al. (1976) observed diploid syncytial nuclei in *Hordeum vulgare* × *H. bulbosum* crosses, presumably as a result of selective elimination of one set of chromosomes. We observed that different events increased the frequency of aneuploidy in oat endosperm. Although concentric (Fig. 1g) or side-by-side spatial separation of genomes likely facilitated the elimination of monogenomic micronuclei (Fig. 1i, j), micronuclei with mixed genome composition were also eliminated (Tomaszewska and Kosina 2021). The micronuclei generally showed a high degree of chromatin condensation (Fig. 1h). The occurrence of multiple bridges (Fig. 1k) and elimination of single chromosomes or their fragments were very common. Additionally, there were several multipolar anaphases (Fig. 1l). However, the pattern of chromosomal elimination in oat syncytial endosperm (Fig. 1g–j; Tomaszewska and Kosina 2021) suggests that the complete uniparental genome elimination at mitosis and interphase seems unlikely in this tissue, and that another cytogenetic or embryological event results in the absence of one haploid set of genomes.

### Embryological analyses of the initial stages of oat endosperm development

**Fig. 2:**
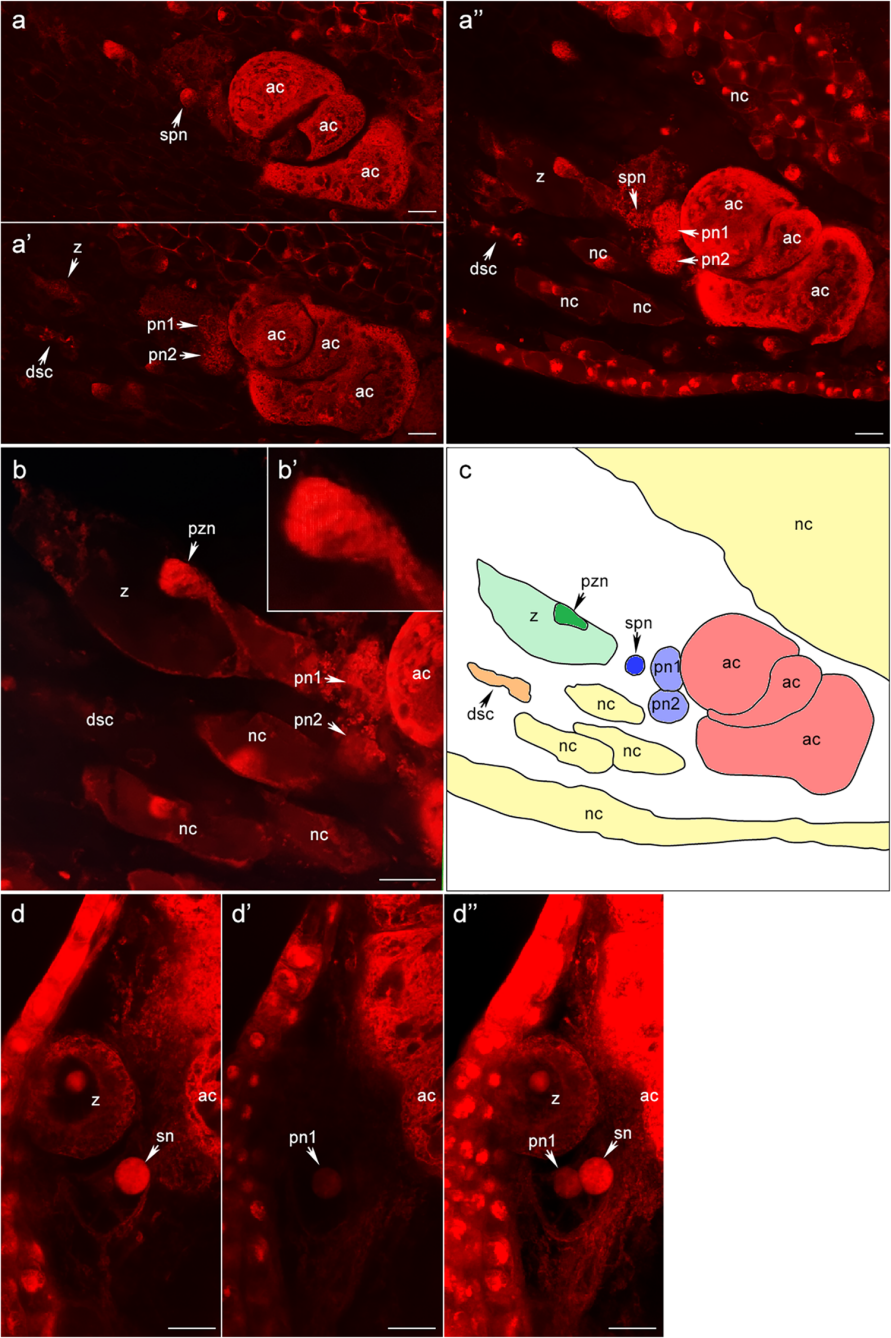
Double fertilisation process in oats. **a**–**c** Early stage of double fertilisation in an amphiploid *A. abyssinica* × *A. strigosa* CIav7423; a–a” serial optical sections from the same embryo sac showing a free sperm nucleus, a zygote, two polar nuclei of the same size, and a degenerating synergid cell; b the same embryo sac as in a–a” with a close-up of the zygote; b’ close-up of the primary zygote nucleus in early prophase; c schematic drawing of the entire embryo sac presented in a–b; **d** later stage of double fertilisation in *A. magna* CIav8331; d–d’ two selected optical sections from the same embryo sac showing a zygote and two polar nuclei differing in size; d” composite diagram from 10 serial sections. Abbreviations according to Portereiko et al. (2006): nc nucellus cell; ac antipodal cell; z zygote, pzn primary zygote nucleus; spn sperm nucleus; dsc degenerating synergid cell; pn1 1st polar nucleus; pn2 2nd polar nucleus; sn secondary nucleus. Scale bars = 20 μm

Observations of the double fertilisation process in a confocal microscope (Fig. 2) confirmed the development of oat endosperm in a different variant than that commonly observed in the monosporic type *Polygonum*. Differences in the volume of sperm (small) and egg (large) nuclei and above all in the degree of chromatin-chromosome condensation in paternal (condensed) and maternal (loose) gametes allow for the correct interpretation of embryological images. Such differences in chromatin condensation were observed in *Nuphar* (Williams and Friedman 2002) and *Amborella* (Friedman and Ryerson 2009). Our embryological analysis of the initial stage of double fertilisation (Fig. 2a–c) revealed a free sperm nucleus (spn), two polar nuclei of the same size (pn1, pn2), and a zygote (z). The absence of one of the two synergids, as well as the presence of one sperm nucleus adjacent to the polar nuclei, indicate that the pollen tube entered the studied embryo sac, consistent with Cannon (1900). The progressive degeneration of the second synergid cell (dsc), the absence of the second sperm nucleus in the embryo sac, and the presence of the primary zygote nucleus (pzn) in early prophase (best seen in Fig. 2b–b’) indicate that the egg cell has been fertilised. Figure 2d shows the later stage of double fertilisation, with a zygote and two polar nuclei of different sizes. The difference in the volume of the two polar nuclei was calculated to be 65%. These data allow us to assume that only one polar nucleus fuses with the sperm nucleus and the other polar nucleus remains free and gives rises to haploid nuclei. This is a different phenomenon from the observed fusion of three haploid nuclei in other cereals.

In our study, the level of pollen viability was higher than 90% and pollen development anomalies were at a level lower than 1% in all the investigated *Avena* accessions. The development of anomalous, multiporate pollen grains was observed only in the case of amphiploids (Tomaszewska and Kosina 2022). Plant development and seed setting in the field cultivation conditions was found to be normal (R. Kosina, unpubl.). Such vigor of offspring indicates generative reproduction with the involvement of both nuclei of sperm cells and results from the embryological events; i.e., the normal double fertilisation of the secondary diploid nucleus is related only to one haploid nucleus of the central cell of the embryo sac, while the second polar nucleus remains free.

As observed in the studies of apomictic grasses (Ma et al. 2009), the development of multiporate pollen grains in oat amphiploids (Tomaszewska and Kosina 2022) would indicate their apomictic reproduction. However, there is no evidence of apomixis and autonomous endosperm development among oats (Bruckner and Hanna 1990). Moreover, the multiporate pollens were noted only in amphiploids among the examined oats, while the domination of the diploid syncytium was observed in the entire group, particularly in the species. Therefore, it can be assumed that anomalous multiporate pollen grains lose the competition with normal pollens due to the slow growth of numerous pollen tubes. It can also be assumed that the formation of multiporate pollens is a consequence of the interaction of different genomes in the nuclei of young allopolyploids, and apomictic reproduction could be some offer for the functioning of polyploid populations with a generative defect (Tomaszewska and Kosina 2022). That does not mean that every species or hybrid producing multiporate pollen is apomictic.

### Correlation analysis

Table 4 shows the results of the correlation analysis of the characters with the addition of two other traits describing anomalous pollen types (see Table 3). The analysis revealed two sets of significantly correlated traits. These traits were related to the ploidy levels of nuclei and the frequencies of anomalous nuclei and the disturbances in the development of pollen grains. The correlation analysis proved that the diploid level of syncytial nuclei was not influenced by anomalous mitoses that produce aneuploid nuclei in endosperm and the increased range of chromosome numbers. The negative coefficients of correlation between the frequency of 2*n* nuclei and the higher ploidy ones (4*n*, 6*n*, hp) indicated that the first group of nuclei changed into the second. The highest coefficient of correlation 0.96*** between the frequency of 4*n* nuclei and that of 6*n* nuclei showed the highest rate of formation of these nuclei from the 2*n* nuclei. The presence of 6*n* nuclei was only observed in a few of the studied taxa (Table 3); however, it can be increased at later stages of endosperm development. The high frequency of aneuploidy and the range of the chromosome numbers correlated with anomalous microsporogenesis, and such interrelations were more frequent in amphiploids (see Tables 2 and 3).

**Table 4:** Pearson’s correlation coefficient matrix of the syncytial endosperm cytogenetic and pollen grain traits (*n* = 15, pooled sample of species and amphiploids)

Traits	1*n*	2*n*	3*n*	4*n*	6*n*	hp	ap	mp	mpp	rcn
1*n*	1.00									
2*n*	0.03	1.00								
3*n*	−0.32	−0.50*	1.00							
4*n*	−0.20	−0.62**	0.51*	1.00						
6*n*	−0.14	−0.68**	0.52*	0.96***	1.00					
hp	−0.28	−0.64**	0.31	0.56*	0.63**	1.00				
ap	0.04	−0.47	−0.38	−0.20	−0.16	0.22	1.00			
mp	0.12	−0.25	−0.47	−0.21	−0.25	0.05	0.81***	1.00		
mpp	−0.16	−0.28	−0.29	−0.16	−0.12	0.31	0.70**	0.73***	1.00	
rcn	−0.12	−0.41	−0.35	−0.02	−0.06	0.21	0.86***	0.76***	0.63**	1.00

1*n*…6*n* levels of ploidy; *hp* hyperploidy; *ap* aneuploidy; *mp* micropollens; *mpp* multiporate pollens; *rcn* range of chromosome number; *, **,*** significance levels at *α*= 0.05, 0.01, 0.001, respectively

### Ordination of taxa in the minimum spanning tree

**Fig. 3:**
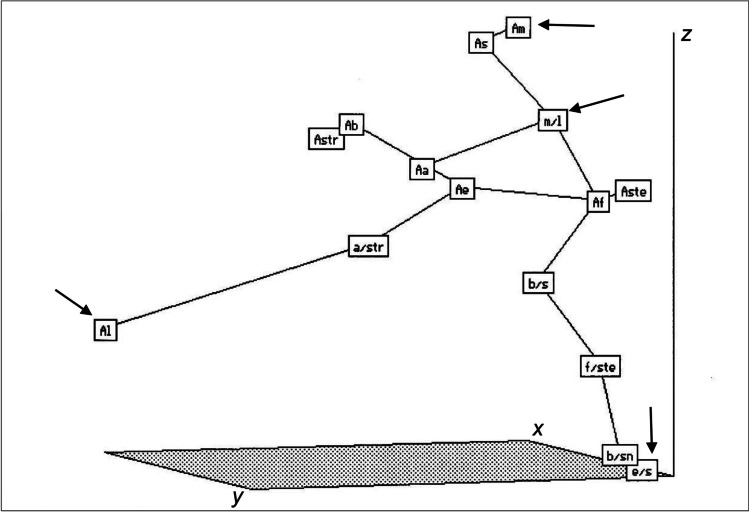
Minimum spanning tree (MST) of amphiploids and parental species (operational taxonomic units, OTUs) of the genus *Avena* in the ordination space (*x*, *y*, and *z* axes) created using Kruskal’s nmMDS method (Rohlf 1994). The OTUs were described by 10 characters presented in Table 4. Abbreviations of OTUs are given in Table 1. The extreme distances between the pairs of OTUs as shown by the MST are maximum a/str-Al 1.194 and minimum Ab-Astr 0.144. Some extreme OTUs are marked with arrows

The species *A. longiglumis* was situated at the extreme point of the ordination space (maximum values of *x* and *y* axes) (Fig. 3). This position was determined by the frequencies of nuclei at all the examined ploidy levels. As a rule, parents showing larger values of ordination axes were well distinguished from amphiploids with smaller values. However, the amphiploid *A. magna* × *A. longiglumis* deviated from this rule, which showed the highest frequency of 2*n* nuclei and the lowest frequency of aneuploid nuclei. This agrees with the data presented in Tables 2 and 3, when a matrix of Euclidean distances between taxa was used as a primary set to put operational taxonomic units (OTUs) in the ordination space.

The numerical taxonomy methods showed clear discrimination between the oat species and the amphiploids in the structural characters of the mature endosperm (Tomaszewska and Kosina 2018) and the features of pollen grains (Tomaszewska and Kosina 2022). These methods also indicated weaker discrimination for the characters listed in Table 4, as these characters describe two different types of events, which were separated in the correlation coefficient matrix. The extreme locations of two species, *A. magna* and *A. longiglumis*, in the ordination space are justified by their characteristics (distributions) in Tables 2 and 3. Data in Table 4 indicate the possible elimination of defective nuclei in the syncytial endosperm, regardless of the ploidy level. Kosina (2016) pointed to the universality of such elimination. This is due to the high variability of the nuclei in the syncytium. The study by Kaltsikes and Roupakias (1975) indicated extreme variability in nuclei shapes in wheat-rye addition and substitution lines. Another study by Bannikova (1975) revealed excellent embryological characteristics with several anomalies in nuclei shapes and the number of nucleoi in a central cell of a hybrid *Hordeum vulgare* × *Secale cereale*. Additionally, the author documented the presence of irregular nuclei, and their condensation and elimination in syncytial endosperm and initially cellularised endosperm in a hybrid *Triticum durum* × *Secale cereale*. The variability of chromosome numbers and ploidy narrowed during the maturation of the endosperm and the elimination of defective structures. The distributions describing the above variability can show platycurtosis (flattening) (Nogler 1984). These show that the development of the endosperm in the genus *Avena* differs from that of the common *Polygonum*-type. Compared to the triploid endosperm, the diploid endosperm has a greater energy potential use in karyokinesis (lower number of chromosomes) and cytokinesis. It is uncertain whether the *Avena* diploid syncytium can transform into a diploid cellular endosperm. In *H. vulgare*, the 3C and 6C nuclei dominated during the syncytium stage of endosperm development, and this condition persisted until the maturity of caryopsis (Nowicka et al. 2021). Other nuclei (12C, 24C), which are the multiples of 3C and 6C, had a lower frequency. These findings demonstrate the stability of the main ploidy level of the endosperm. However, the analyses of DNA content in young cells of the aleurone layer in *A. fatua* confirmed the levels of 3C and 6C, as well as the presence of 2C nuclei among nuclei within a large range of DNA content at the stage of full maturity of the aleurone layer (Maherchandani and Naylor 1971). Among the species listed in Table 3, *A. fatua* has a higher frequency of 1*n* and aneuploid nuclei. This explains the variation in DNA levels to a certain extent, but the appearance of 2C nuclei in the aleurone layer is interesting.

## Conclusions

It has been shown that the chromosome numbers in the syncytial endosperm greatly vary for a wide range of oats, species, and amphiploids. This is also reflected in the shapes of the trait frequency distributions. However, diploid nuclei are the most dominant group. This cytogenetic pattern is common regardless of the level of ploidy of species and diversity of amphiploids. Therefore, it can be assumed that this applies to the whole *Avena* genus. Diploid syncytium, as well as subsequent cellular endosperm, arises when the polar nuclei of the central cell are not fused, and one of them fuses with the sperm nucleus. The 1*n* nuclei likely arise from a free polar nucleus. The fusion of dumbbell nuclei with a lower DNA content results in the formation of nuclei with higher levels of ploidy. Defective structures, such as nuclei, chromosomes, their fragments, and micronuclei, with a different DNA content than 1*n* and its multiples, increase the frequency of aneuploidy. The phenomenon of aneuploidy does not correlate with a basic syncytial ploidy, but correlates with anomalous pollen development. The dominance of diploid nuclei in the oat endosperm can be thoroughly understood by performing advanced embryological analyses. The results presented in this paper show that the oat endosperm does not develop according to the common *Polygonum*-type embryo sac but its modification.
